# The effectiveness of health-oriented leadership interventions for the improvement of mental health of employees in the health care sector: a systematic review

**DOI:** 10.1007/s00420-020-01583-w

**Published:** 2020-10-04

**Authors:** Felicitas Stuber, Tanja Seifried-Dübon, Monika A. Rieger, Harald Gündel, Sascha Ruhle, Stephan Zipfel, Florian Junne

**Affiliations:** 1 Department of Psychosomatic Medicine and Psychotherapy, University Hospital Tuebingen, Tuebingen, Germany; 2 Institute of Occupational Medicine, Social Medicine and Health Services Research, University Hospital Tuebingen, Tuebingen, Germany; 3 Department of Psychosomatic Medicine and Psychotherapy, Ulm University Medical Center, Ulm, Germany; 4 Department of Business Administration, in particular, Work Human Resource Management and Organization Studies, Heinrich-Heine-University Düsseldorf, Düsseldorf, Germany

**Keywords:** Leadership, Intervention, Mental health, Health care sector, Employees, Prevention

## Abstract

**Purpose:**

An increasing prevalence of work-related stress and employees’ mental health impairments in the health care sector calls for preventive actions. A significant factor in the workplace that is thought to influence employees’ mental health is leadership behavior. Hence, effective leadership interventions to foster employees’ (leaders’ and staff members’) mental health might be an important measure to address this pressing issue.

**Methods:**

We conducted a systematic review according to the PRISMA statement (Liberati et al. 2009) and systematically searched the following databases: PubMed (PMC), Web of Science, PsycINFO (EBSCOhost), EconLit (EBSCOhost), and Business Source Premier (EBSCOhost). In addition, we performed a hand search of the reference lists of relevant articles. We included studies investigating leadership interventions in the health care sector that aimed to maintain/foster employees’ mental health.

**Results:**

The systematic search produced 11,221 initial search hits in relevant databases. After the screening process and additional literature search, seven studies were deemed eligible according to the inclusion criteria. All studies showed at least a moderate global validity and four of the included studies showed statistically significant improvements of mental health as a result of the leadership interventions.

**Conclusions:**

Based on the findings, leadership interventions with reflective and interactive parts in group settings at several seminar days seem to be the most promising strategy to address mental health in health care employees. As the available evidence is limited, efforts to design and scientifically evaluate such interventions should be extended.

## Introduction

On one hand, mental health can be seen as a basic human need that influences the individual quality of life in general. On the other hand, mental illnesses cause a large economic loss worldwide. For example, Patel et al. (2018) estimated the global economic loss due to mental illnesses between 2010 and 2030 at US$ 16 trillion worldwide. Thus, mental health may be considered an important variable concerning ethical and economic aspects in the modern working world.

In this review, the term mental health is defined according to the conceptualization of the World Health Organization (World Health Organization 2001), which describes *mental health* as a continuous variable ranging from a negative, symptom-based pole to a positive pole concentrating on psychological functioning. In detail, the term mental health can be conceptualized as being based on negative symptoms such as psychological harm and pathologies like depression, burnout, and their related physical symptoms (e.g., sleeping disorders). It can also be conceptualized as positive mental health in the form of emotional, psychological, and social well-being (Montano et al. 2017; Westerhof and Keyes 2010).

Considering both sides of mental health, its maintenance in working contexts is no longer seen only as an employee’s individual task. Rather, political stakeholders as well as scientists increasingly discuss the issue of prevention in mental health as an organizational task; that is, the organization and its representatives, especially leaders, are seen to have the responsibility for upholding their employees’ mental health (e.g., Thomas et al. 2018; WHO Regional Committee for Europe 2013). This is in concordance with occupational health and safety regulations emphasizing the enterprise’s responsibility to avoid or minimize all kinds of work-related risk factors (Council of the European Communities 1998).

This extension from individual to common organizational responsibility can be seen of especially high importance in psychologically and physiologically demanding working contexts such as the health care sector. For example, Zhou et al. (2017) found the highest rate of “work-related mental ill health” (p. 310) for nurses, followed by ambulance staff and physicians compared to social workers and teachers working within the social sector in the UK.

The higher prevalence of mental illnesses in health care employees (for an overview, see Harvey et al. 2017) might be partly explained by the difficult working conditions that characterize the work in the health care sector (Harvey et al. 2017). Besides an increased workload and staff shortage (Royal College of Physicians 2016), studies showed an effort-reward imbalance (Schulz et al. 2009; Weyers et al. 2006); that is, employees perceived an imbalance between the effort they put into their work and the reward they obtained for it (e.g., salary, appreciation). Furthermore, physicians have reported that their workplace is characterized by high job demands but low job control (Bauer and Groneberg 2015). And Kivimäki et al. (2003) found that amongst hospital employees, low procedural justice, for example when processes are perceived as intransparent and non-participative, was linked to a higher risk of sickness absence in relation to high procedural justice. Finally, health care workers state to have high psychological burdens in their daily work (Bernburg et al. 2016) and can be confronted with acute crises which cause incredible psychological stress such as serious accidents with lots of heavily injured patients or pandemics like COVID-19 (Zhu et al. 2020).

Taken together, health care workers can be seen as a group with special working conditions which may lead to a large amount of work stress and can in turn promote the development of certain mental disorders. Furthermore, the growing strain in health care professions (e.g., physician burnout affects over 50% of physicians in the USA) can also be seen as a danger for patient safety (The Lancet 2019).

An important factor that can buffer at least some negative aspects of the mentioned working conditions on staff members’ mental health is leadership behavior. In more detail, leadership behavior is an important working condition in day-to-day work that has been associated with staff members’ mental health in both positive and negative ways. Destructive leadership behavior is negatively associated with well-being (Schyns and Schilling 2013), and a lack of supportive leadership decreased self-rated health in men even ten years later (Schmidt et al. 2018). From a positive perspective, Finne et al. (2014) reported in their prospective panel study that fair leadership behavior and the support of direct supervisors are the most protective factors for staff members’ mental health.

**Fig. 1:**
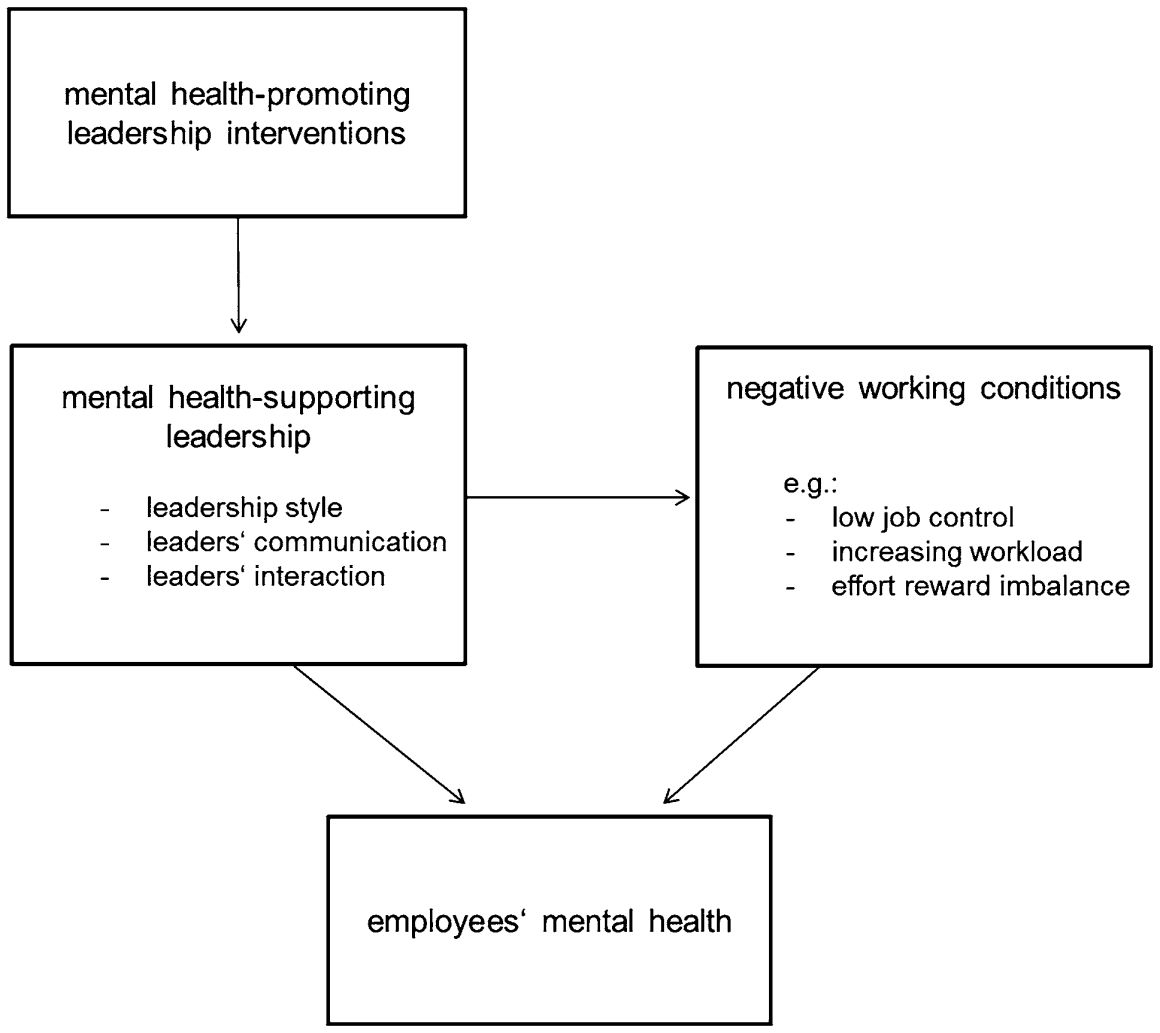
Relationship between leadership training and staff members’ mental health

Based on the health-oriented leadership concept (HoL) of Franke et al. (2014) health-oriented leadership can be defined as a general term to describe a behavioral and organizational health-preventive approach consisting of ‘leader-centered’ and ‘staff-centered’ aspects. Leader-centered aspects include the mindsets, attitudes/beliefs and behaviors of leaders, which influence the leaders’ own health behavior and stress experience. Whether the leader her/himself is under stress is one important factor for staff members’ health, as it can spill over indirectly because of the leaders’ role model function, or directly through leadership behavior communication or interaction, to staff members’ mindsets, attitudes/beliefs and behaviors (Elprana et al. 2016; Franke and Felfe 2011). As a consequence, leaders’ own health is an important factor in health-oriented leadership. Staff-centered aspects of health-oriented leadership comprise the creation of a mental health-promoting work conditions (e.g., Nielsen et al. 2008) as well as direct attentive communication and interaction with staff members (e.g., proactively addressing stressed staff members to find solutions or help with prioritizing work tasks) in a participative process (Elprana et al. 2016; Franke and Felfe 2011). To sum up, a health-oriented leader pays attention to her or his own physical and psychological health (behavior prevention) and addresses the health of staff members through her or his communication, leadership behavior, and as a role model (organizational prevention, Skakon et al. 2010, see Fig. 1). When we refer to health-oriented leadership in this manuscript, we not necessarily mean the HoL concept in the strict sense as it was drawn up by Franke et al. (2014) but rather in a broader sense encompassing all leadership behavior that has the health of employees as a longterm goal.

Montano et al. (2017) emphasizes the future need for leadership interventions from an occupational health point of view. This is especially true for psychologically and socially demanding workplaces such as those in the health care sector. Leadership plays an important role in emergency situations, in the establishment of team play, high-quality inter-professional cooperation and daily work with serious ill patients and can help to prevent psychological illnesses of health care workers not least to secure the medical care of patients. With regard to the health care sector, staff-centered leadership behaviors, which is as well helpful in other work sectors, such as the leader-member-exchange model (LMX; for an overview see Graen and Uhl-Bien 1995), transformational leadership behavior (Bass 1999; Podsakoff et al. 1996) or servant leadership (Blanchard 2018) have been shown to be associated with improved mental health (Eva et al. 2019; Gregersen et al. 2014). Some cross-sectional studies point to the positive correlations of health-oriented leadership for staff members. For example, transformational leadership goes along with increased job satisfaction and less workplace absenteeism in nurses (Boamah et al. 2018; Lee et al. 2011).

When contrasting the potential psychological strain of the workplace *health care sector* and the potential health-maintaining and promoting aspects of leadership behavior, international experts have recently begun to support clinical leadership interventions with a focus on leaders’ communication (e.g., giving feedback to staff members), interaction (e.g., nonverbal communication or fostering team work) and leadership style (e.g., transformational leadership) to promote a healthier workforce (Saravo et al. 2017). Leadership interventions are, therefore, an important instrument to be aware of, learn and practice health-oriented leadership. To emphasize the importance of health-oriented interventions Wijnen et al. (2020) showed that stress reducing interventions among health care workers improved staffs' productivity on a monetary level and showed a 60-fold payout. However, too persuade top management in the health care sector to implement health oriented leadership interventions, such systematic evidence of effectiveness is needed. Yet, a systematic approach is missing and the scattered knowledge does currently not provide a clear picture regarding the advantages of health-oriented leadership interventions. To target this gap, the first step should be an overview of leadership interventions’ (psychologically and economically) effectiveness, in particular an overview of the effectiveness of longitudinal studies (at least with a measurement point before and after the intervention), as they show the possible change potential regarding employees’ mental health and thereby contribute to the improvement of health care quality.

Hence, our aim was to record the existing longitudinal studies regarding the effectiveness of leadership interventions towards mental health in the health care sector. Since health-oriented leadership is a concept with many facets, we focused on leadership interventions that target communication as a leadership tool, interaction as a relationship-oriented factor, or leadership style as specific leadership concepts. With this limitation, we were able to focus on leadership as an occupational health factor.

The research question of this review was therefore formulated as follows:

How do interventions that target leadership in the health care sector with a focus on communication, interaction or leadership style influence the mental health of leaders and/or of their staff members working in the health care sector?

By doing so, we provide accumulated knowledge about leadership interventions including their dose, content and target group in the health care sector as one contribution to inform other researchers in the field how to design future studies which ultimately may strengthen the evidence on the effectiveness of such interventions.

## Methods

The systematic review was conducted according to the PRISMA guidelines (Preferred Reporting Items for Systematic Reviews and Meta-Analyses; Liberati et al. 2009; Moher et al. 2009). The reporting of methods in the following is structured according to the PRISMA checklist (Liberati et al. 2009, p.3).

### Registration

After developing a research protocol, the systematic review was registered at the International Prospective Register of Systematic Reviews (PROSPERO) of the National Institute for Health Research (NHS). The registration is available under no. CRD42018088632 at www.crd.york.ac.uk/prospero/display_record.php?RecordID=88632. The registration took place after the search strategy and the databases were decided on and before the screening process was initiated.

### Eligibility criteria

We applied the PICOS criteria (Participants, Intervention, Comparator, Outcome, Study Design; Liberati et al. 2009; Moher et al. 2009) described in Table 1 to select studies in a standardized manner to answer our research question. In detail, PICOS criteria were utilized to develop our search strategy as well as to select studies in the screening process, and they guided the structured full-text analyses of included studies.

**Table 1:** Applied PICOS criteria

PICOS criteria	Inclusion	Exclusion
Participants	Leaders and/or staff members working in the health care sector	Leaders or staff members working outside the health care sector
Intervention	A leadership intervention to improve or maintain leaders’ or staff members’ mental health, by building or shaping leadership style, communication or interaction skills Intervention typ: face-to-face interventions, online interventions, handouts, supervision, intervision, coaching, case conferences, or academic training programs	Interventions only for staff members (employees without leadership responsibility)
Comparator	Possible but not required	
Outcome	Indicator of mental health in leaders and/or staff members (e.g., stress, well-being, burnout, affective symptoms, physical health problems corresponding to mental health e.g., chronic pain) Measured by subjective measurements (e.g., questionnaires, qualitative data like video and audio, participating or non-participating observation) or objective measurements (e.g., number of sick days, number of department changes inside one organization, number of resignations, physiological measurements of mental health like heart rate or cortisol level)	No indicators/outcomes of mental health in leaders or staff members Studies that do not measure any mental health outcome
Study design	Measurement of a mental health indicator at least twice, with one time point before and one time point after the administration of the intervention with and without control group	Studies that only measure one time point Case studies

### Search

We searched psychological, medical and economic electronic databases, namely PubMed (PMC), Web of Science, PsychINFO (EBSCOhost), EconLit (EBSCOhost), and Business Source Premier (EBSCOhost), from inception to 16 May, 2018 and updated our search until 27 May, 2019. The search strategy was developed in a discursive group process by means of the PICOS criteria and followed this general scheme: content AND intervention AND outcome AND setting AND outcome assessor for each of the core concepts included a variety of keywords. As an example, the search strategy for the PubMed database was: (leadership OR communication OR interaction) AND (intervention OR training OR education OR skills OR prevention OR program OR curriculum OR “skill enhancement” OR “vocational training” OR “vocational trainings” OR “on-the-job-training” OR “on-the-job-trainings” OR “leadership training” OR “leadership trainings”) AND (“mental health” OR “psychological health” OR “psychological strain” OR “mental strain” OR “stress” OR ”well-being” OR “stress reduction” OR “stress prevention”) AND (hospital OR clinic OR “general practice” OR “general practices” OR "private practice" OR “private practices” OR “medical practice” OR “medical practices” OR “inpatient service” OR “inpatient services” OR “outpatient service” OR “outpatient services”) AND (doctor OR physician OR “practitioner” OR “practitioners” OR nurse OR “doctor’s assistant” OR “doctor’s assistants” OR “medical assistant” OR “medical assistants” OR employee OR worker OR workforce OR follower OR “group member” OR “group members” OR staff OR subordinate OR manager OR leader). The search strategies for the other databases were similar with a few changes to accommodate database-specific requirements. For the searches in PsycINFO, EconLit and Business Source Premier, we applied the advanced search filters “apply related words” and “apply equivalent subjects” and “Academic Journals”. We decided to include published original articles in English and German.

Title and abstract of the electronically selected studies were screened by two independent raters according to the inclusion criteria to avoid the rejection of relevant studies. After the screening process, we further examined all studies that had been include by at least one rater for eligibility via full-text analyses and supplemented the identified studies by a hand search of the reference lists of the included studies.

### Data preparation

The content of the included articles was extracted in a standardized procedure based on the PICOS criteria. The small number of eligible studies, together with a high level of heterogeneity, hindered meta-analytic processing of the available evidence. Instead, we employed a narrative approach. The following dimensions were extracted: countries, where the intervention took place, setting of the intervention (organization), intervention group (e.g., hierarchy level, number of participants), control group (if applicable), intervention type (e.g., coaching, workshop, or supervision), dose/duration of intervention (i.e., how often and how long the intervention was administered), content/reference frame of the intervention (i.e., concepts or leadership styles taught in the intervention), time points of measurement (e.g., before and after the intervention, and/or follow-up measurement), type of measurements (i.e., qualitative, quantitative or mixed method), outcomes (i.e., utilized questionnaires), target group (i.e., group from whom outcome measurements were collected), and evidence for effectiveness of leadership intervention. Any uncertainties during data extraction and preparation were resolved through discussion between the authors.

Besides the content-related analyses, we assessed the validity of the eligible studies by the Quality Assessment Tool for Quantitative Studies, developed by the Effective Public Health Project (Effective Public Health Practice Project 2007; Thomas et al. 2004) as recommended in the Cochrane Handbook for Systematic Reviews of Interventions (Armstrong et al. 2008). The six quality categories (selection bias, study design, confounders, blinding, data collection method) as well as withdrawals and drop-outs, can be judged as ‘weak’, ‘moderate’ or ‘strong’ by this tool. Two raters assessed the risk of bias of the seven studies independently. Any rating discrepancy was resolved through discussion in the study group.

## Results

### Study selection

**Fig. 2:**
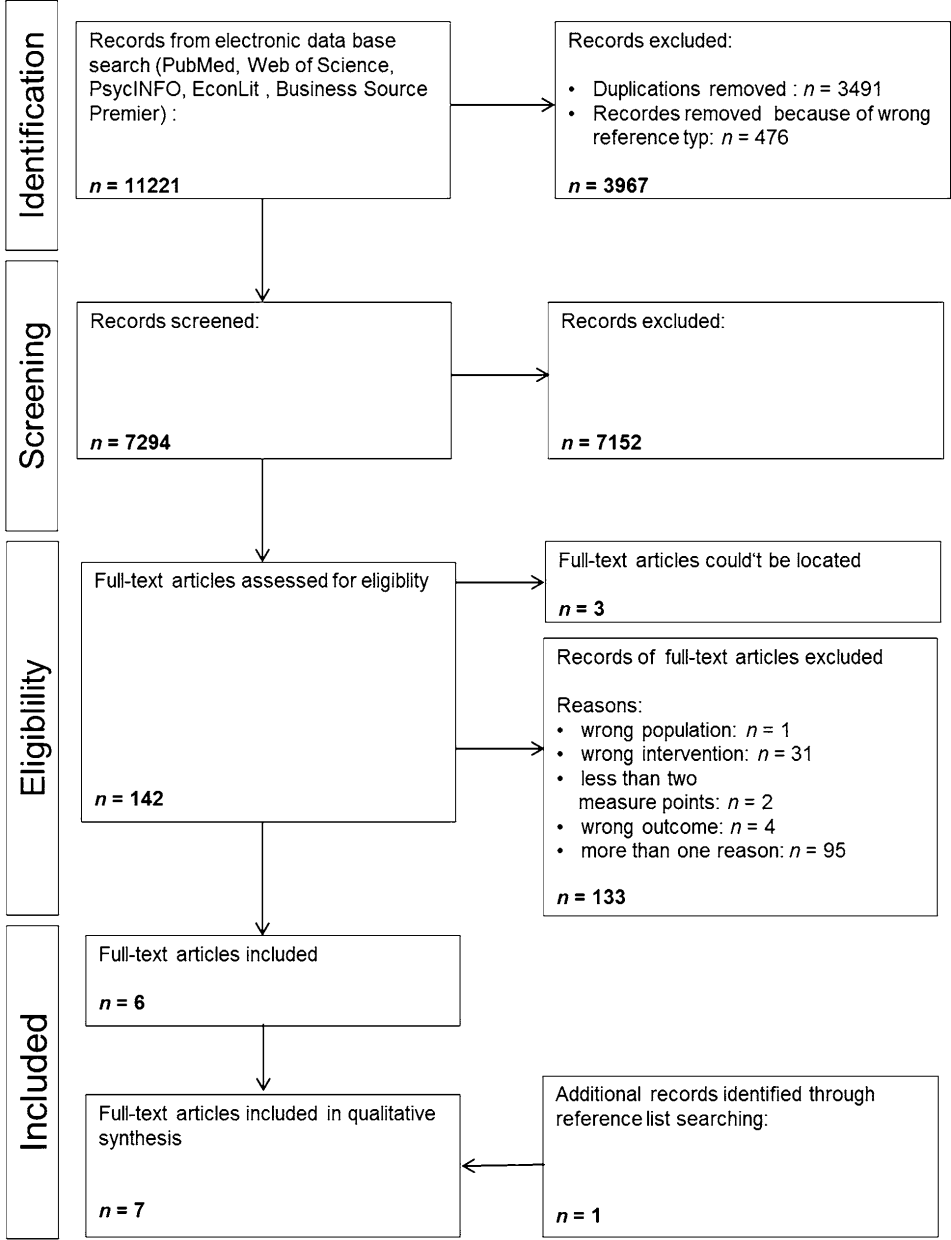
Flowchart of study selection

We identified 11,221 hits in the relevant electronic databases from inception until the last update of the search (27th May, 2019). After removing duplicates, items with unfitting study format for this purpose (e.g., reviews, meta-analyses, book chapters, case studies) and hits with unknown authorships, the titles and abstracts of the remaining 7294 original articles were screened by two independent raters based on the PICOS criteria. Overall, 142 articles were included for full-text analysis by at least 1 rater. Three articles that were not available online and not accessible by either contacting the corresponding article authors or different article delivery services were deemed unattainable. Based on the remaining 139 articles, we conducted a full-text screening as well by means of the PICOS criteria. After the full-text screening, any uncertainties in the evaluation were discussed within the interdisciplinary author team. Thereafter, 6 articles were left from the database search, whereas 133 articles were excluded because of not meeting the inclusion criteria in terms of nature of the population, means of the intervention content, and less than 2 measurement time points, regarding the outcome or 2 or more of these issues. For a detailed description of the selection analysis, see Fig. 2.

Beyond the electronic database search, we conducted a reference list hand search consisting of the five eligible articles, relevant literature on leadership as well as thematically linked reviews and meta-analysis (including those that were hits in our electronic search). Eventually, one additional article was selected, so in the end, seven articles fully met the inclusion criteria and were subjected to the full-text analyses and quality assessment procedures (for the PRISMA flowchart, see Fig. 2).

### Study quality of quantitative study parts

Quantitative studies in psychological health care research or assessing the effects of psychological preventive measures often endeavor to develop or display (new) forms of health care or psychological offers that improve subjective psychological variables and can be transferred directly into practical work. That is, some quality assessment criteria, such as *blinding* or *confounders,* cannot be applied without caution. For example, in this systematic review, six of the seven eligible studies were based on self-evaluation through psychological questionnaires, which made blinding impossible. This is also true for the avoidance of potential confounders (e.g., gender imbalance) which cannot be influenced because they are immanent factors of the health care sector (World Health Organization 2008) and cannot be controlled in field studies. Keeping that in mind, all included studies showed a high risk of potential bias, but the results of the validity assessment showed that almost every included study described the quality components confounders and blinding insufficiently. When disregarding these two components, all studies showed at least a moderate global validity rating. For more details, see Table 2.

**Table 2:**
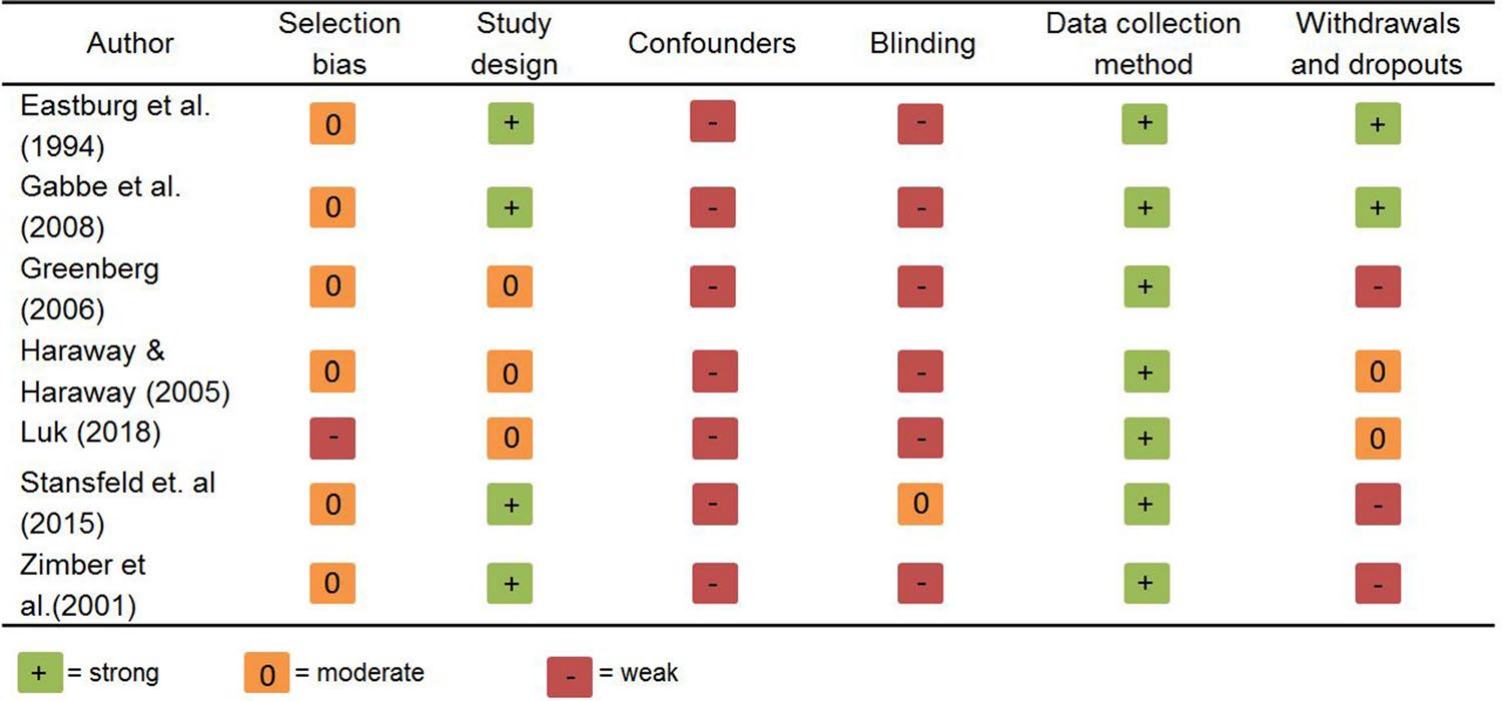
Study quality of the quantitative parts of the eligible studies

### Study characteristics

The majority of the included studies were presented in English language and conducted at hospitals/health care institutions (Eastburg et al. 1994; Greenberg 2006; Haraway and Haraway 2005; Luk 2018; Stansfeld et al. 2015). One study was implemented at a medical university (Gabbe et al. 2008) and one in a retirement home in Germany (Zimber et al. 2001). Five interventions were designed for only one specific occupational group: four studies addressed nursing staff (Eastburg et al. 1994; Greenberg 2006; Luk 2018; Zimber et al. 2001), and one addressed new chairs of the department of obstetrics and gynecology (Gabbe et al. 2008), whereas two interventions were interprofessional (Haraway and Haraway 2005; Stansfeld et al. 2015), that is, all occupational groups of the organization could participate. Overall, a total of 191 leaders took part in an intervention on leadership, communication or interaction topics. However, in one study, the exact number of participating leaders was not mentioned (Eastburg et al. 1994). All studies but two (Haraway and Haraway 2005; Luk 2018) chose a controlled design in the form of a controlled clinical trial (Eastburg et al. 1994; Stansfeld et al. 2015; Zimber et al. 2001), a cohort analytic design (Greenberg 2006) and a randomized controlled trial (Gabbe et al. 2008). Mental health was measured in a total of *n* = 648 staff members and *n* = 86 leaders. The outcome measures differed across the studies: three studies assessed changes in mental health in staff members only (Eastburg et al. 1994; Greenberg 2006; Stansfeld et al. 2015), three studies in leaders only (Gabbe et al. 2008; Haraway and Haraway 2005; Luk 2018), and one study measured changes in both hierarchy levels (Zimber et al. 2001). For more details, see Table 2.

### Longitudinal measurements: time points and outcomes

Mental health was measured quantitatively before leadership interventions and at one to three time points after the interventions in all included studies. The measurement point after the intervention differed from directly after the intervention (Zimber et al. 2001) to up to one year after the start of the intervention (Haraway and Haraway 2005). Additionally, Haraway and Haraway (2005), Luk (2018), and Stansfeld et al. (2015) supplemented the quantitative measurement with a qualitative approach mainly not only to assess acceptance, feasibility and potential improvements of the intervention, but also to reflect on the intervention content with regard to leaders’/staff members’ health. Since these qualitative measurements only support the use of the quantitative measures in all of the three studies, we focus on the quantitative data when answering the current research question.

All outcome assessors evaluated their own subjective mental health by standardized subjective measurements in the form of questionnaires. Stansfeld et al. (2015) additionally measured sickness leave on an organizational level as an objective variable. As far as the measured constructs are concerned, mental health was either operationalized by one single outcome (e.g., insomnia by Greenberg 2006) or rather broadly by a variety of outcomes (e.g., well-being, psychological distress, self-reported sickness absence by Stansfeld et al. 2015). Six studies conceptualized mental health as the absence of psychological strain. As variables of psychological strain, e.g., burnout, stress, insomnia or sickness leave were measured, whereas in two studies, mental health was evaluated as the presence of well-being. For more details, see Table 2.

### Intervention: content and effects

The seven included studies were considerably heterogeneous concerning type, dose and content of the administered leadership intervention. Four of the included studies were structured as group interventions (workshops) with a total duration between 4 and 21 h. In the other three studies, the intervention was delivered on an individual basis with a total duration between 1 h and a flexible time (Eastburg et al. 1994; Gabbe et al. 2008; Stansfeld et al. 2015). As far as the content of the interventions is concerned, studies addressed staff-centered outcomes: leadership skills, which may improve the collaboration with staff members, as well as leader-centered outcomes: skills that may support leaders in their own mental health behavior and stress prevention. Three studies focused on staff-centered outcomes (e.g., giving positive feedback; Eastburg et al. 1994; Greenberg 2006; Haraway and Haraway 2005). Three studies were multimodal with staff-centered as well as leader-centered contents (Luk 2018; Stansfeld et al. 2015; Zimber et al. 2001) and lastly, the peer-mentor program delivered by Gabbe et al. (2008) was completely individual, and the content was not transparent.

Out of the three staff-centered interventions, Eastburg et al. (1994) conducted a one-hour, one-to-one, psychoeducational intervention on positive feedback, with the main focus on the reflection of leaders’ feedback skills and the transmission of positive feedback in the daily routine. A standardization of the intervention was not described. With reference to the results, an intervention effect could be shown for one sub-dimension of burnout (Maslach and Jackson 1981). In particular, the nursing staff of trained leaders showed a decrease in *emotional exhaustion* compared to the control group (*F*(1,2.99), *p* < 0.05, pre/postchange score intervention group: − 1.29, pre/postchange score control group: 1.90). The intervention and control group did not differ in their burnout ratings. Thus, mean and standard deviation were only presented for intervention and control group together (emotional exhaustion: *M* = 19.1, *SD* = 11.1; depersonalization: *M* = 6.8, *SD* = 5.7; personal accomplishment: *M* = 36.8, *SD* = 8.2).

The second staff-centered study by Greenberg (2006) took place in four hospitals of one large health care organization. In half of the hospitals, the pay system for nurses changed in the study period so the nurses at these two hospitals got less salary than before. The salary changes were a quasi-experimental manipulation without any researcher involvement. The researcher only knew about the payment change earlier than the employees. For the intervention, one hospital with salary change (IG_underpaid_) and one hospital without the salary change (IG_no payment change_) participated in the intervention group. The control group composed as well of a hospital with salary change (CG_underpaid_) and a hospital without salary change (CG_no payment change_). The intervention was conducted after the salary change and consisted of a standardized leadership training on organizational justice with a main focus on interactional justice (Skarlicki and Latham 2005) with theoretical and practical parts as well as discussion groups. Leaders learned how to provide information and give emotional support to their staff members. The self-reported insomnia was recorded at four time points (before salary change (T1), after salary change and before leadership training (T2), direct after the leadership intervention (T3) and 6 months after the leadership intervention (T4)). The self-rated insomnia showed an intervention x payment x time interaction *F*(3,1386) = 9.99, *p* < 0.01, *η*^*2*^ = 0.02. At T2, T3 and T4, nurses with no payment change differed from nurses with payment change statistically significant in their reported insomnia (T2: *M*_no payment change_ = 2.58, *SD* = 1.10; *M*_underpaid_ = 5.85, *SD* = 0.90; *F*(1,465) = 1,184.04, *p* < 0.01, *η*^*2*^ = 0.72, T3: *M*_no payment change_ = 2.76, *SD* = 1.13; *M*_underpaid_ = 5.07, *SD* = 1.19; *F*(1, 465) = 460.29, *p* < 0.01, *η*^*2*^ = 0.50, T4: *M*_no payment change_ = 2.77, *SD* = 1.07; *M*_underpaid_ = 4.29, *SD* = 1.40; *F*(1, 465) = 176.65, *p* < 0.01, *η*^*2*^ = 0.28). The intervention showed an effect on the self-reported insomnia of the underpaid nurses. At T3 and T4, all four groups differed statistically significant (T3: *F*(3, 463) = 206.84, *p* < 0.01, *η*^*2*^ = 0.57, T4: *F*(3,463) = 92.84, *p* < 0.01, *η*^*2*^ = 0.38). The underpaid nurses with trained leaders reported less insomnia than the underpaid nurses with untrained leaders directly after the organizational justice training as well as 6 months later.

Lastly, Haraway and Haraway (2005) set their staff-centered focus on conflict management (e.g., development, reaction and resolution of conflicts) as well as on communication skills and a standardized training on leading difficult subordinates; developed by Bissell (1993). However, they assessed only the leaders’ self-reports of work-related stress. Specifically, participating leaders stated significantly lower occupational stress in the four sub-areas role overload (*M*_pretest_ = 56.39, *SD*_pretest_ = 8.90; *M*_posttest_ = 52.61, *SD*_posttest_ = 10.43; *t* = 2.33, *p* = 0.03), interpersonal strain (*M*_pretest_ = 50.43, *SD*_pretest_ = 8.16; *M*_posttest_ = 46.52, *SD*_posttest_ = 8.14; *t* = 2.65, *p* = 0.02), role boundary (*M*_pretest_ = 55.13, *SD*_pretest_ = 10.39; *M*_posttest_ = 51.39, *SD*_posttest_ = 11.81; *t* = 2.57, *p* = 0.02), and psychological strain (*M*_pretest_ = 52.09, *SD*_pretest_ = 9.97; *M*_posttest_ = 48.61, *SD*_posttest_ = 8.18; *t* = 2.51, *p* = 0.02).

Luk (2018), Stansfeld et al. (2015) and Zimber et al. (2001) took a multimodal leadership approach. Luk (2018) conducted an intervention to foster the reflection and development of personal and professional attitudes, values and skills in the sense of servant leadership as well as a part of stress reduction skills for the leaders. Therefore, the participating nursing leaders learned about leader-centered aspects such as self-care and resilience in nursing and ‘staff-centered’ aspects such as sharing leader experience or managing difficult staff members. The leadership intervention was divided into three different parts: a seminar part, a group sharing part and a 1-day retreat. In a pre-post comparison, participants showed statistically significant improvements in servant leadership and workplace well-being. In more detail, the overall score of servant leadership (*M*_pretest_ = 3.61, *SD*_pretest_ = 0.30; *M*_posttest_ = 3.85, *SD*_posttest_ = 0.38; *t*(25) = 4.03, *p* < 0.001) as well as the subscales of servant leadership: empowering staff members (*M*_pretest_ = 3.63, *SD*_pretest_ = 0.50; *M*_posttest_ = 3.87, *SD*_posttest_ = 0.58; *t*(25) = -2.07, *p* = 0.049), behaving ethically (*M*_pretest_ = 3.96, *SD*_pretest_ = 0.42; *M*_posttest_ = 4.15, *SD*_posttest_ = 0.39; *t*(25) = -2.30, *p* = 0.03), having conceptual skills (*M*_pretest_ = 3.81, *SD*_pretest_ = 0.43; *M*_posttest_ = 4.06, *SD*_posttest_ = 0.36; *t*(25) = -2.39, *p* = 0.025), creating values for those outside of organization (*M*_pretest_ = 2.92, *SD*_pretest_ = 0.91; *M*_posttest_ = 3.52, *SD*_posttest_ = 0.77*; t*(25) = -3.92, *p* = 0.001) showed significant improvements. Is also applies for the overall workplace well-being (*M*_pretest_ = 2.48, *SD*_pretest_ = 0.37; *M*_posttest_ = 2.70, *SD*_posttest_ = 0.29; *t*(25) = -3.76, *p* = 0.001.) and its’ subscales: work satisfaction (*M*_pretest_ = 2.72, *SD*_pretest_ = 0.42; *M*_posttest_ = 3.02, *SD*_posttest_ = 0.39; *t*(25) = -3.39, *p* = 0.002), organizational respect for the employee (*M*_pretest_ = 2.46, *SD*_pretest_ = 0.45; *M*_posttest_ = 2.77, *SD*_posttest_ = 0.37; *t*(25) = -3.28, *p* = 0.003) and employer care (*M*_pretest_ = 2.43, *SD*_pretest_ = 0.61; *M*_posttest_ = 2.77, *SD*_posttest_ = 0.47; *t*(25) = -3.06, *p* = 0.005).

Stansfeld et al. (2015) addressed topics that were rather leader-centered e.g., stress management such as dealing with stress sources, understanding the link between mental and physical health, leaders’ legal duty of care and their leadership style as well as rather staff-centered topics such as supporting staff members and teams in problem-solving, find individual staff-centered solutions, on staff member and team level. To this end, they utilized a standardized e-learning program for leaders (Anderson Peak Performance package, https://www.andersonpeakperformance.co.uk) in a mainly online-based approach. However, the e-learning leadership intervention showed no significant effect. Staff members reported no significant changes in any investigated indicator of mental health.

In the third multimodal approach, Zimber et al. (2001) concentrated their group intervention for leaders and staff members on the following topics without referring to a standardized manual: coping with ‘difficult’ residents, coping with personal stress, communication with staff members, and leadership style. Leaders and staff members participated together in two-thirds of the intervention, whereas one-third of the intervention was delivered separately. However, the study results were presented together for leaders and staff members, and therefore, leadership-specific changes in either leaders themselves or staff members could not be assessed. Significant improvements in the intervention group compared to the control group from the first to the second measurement time point were only found in relationship to residents but not in mental health-related outcomes. Nevertheless, changes in personal competences from before until 3–4 months after the intervention could predict a significant amount of variance in working strain (*R*^*2*^ = 0.33, *F* = 6.4, *p* < 0.001) and psychological impairment (*R*^*2*^ = 0.32, *F* = 6.2, *p* < 0.001).

Gabbe et al. (2008) implemented an individual, 1-year peer-mentoring program between new chairs of obstetrics and gynecology departments and experienced chairs. The authors had no concrete requirements for the participating chairs concerning what content should be mentioned in their peer-mentoring contacts except that the intervention should support the new leaders by developing the necessary skills to be successful as a chair. The authors observed no differences in perceived burnout symptoms between participating chairs and control group before and after the peer-mentoring program. For more details, see Table 3.

**Table 3:** Characteristics of included studies

Author	Setting	Study design	Sample (IG/CG;n)	Outcome assessors (IG/CG;n)	Intervention type; dose	Content of the intervention	Measure points	Type of measurement	Results
Eastburg et al. (1994)	USA, 1 private medical hospital	Controlled clinical trial	Nursing leaders (IG; number not mentioned), (CG; number not mentioned)	Nursing staff members (IG; 34), (CG; 28)	One-to-one meeting with a researcher; 1 × 1 h	Positive feedback and its relation to staff members’ mental health, transfer of positive feedback into daily routine	t0: before the intervention; t1: 30 days after the intervention	Quantitative; Self-rated burnout (Maslach Burnout Inventory, MBI; Maslach and Jackson 1981)	t0–t1: emotional exhaustion, ↓ in IG (IG vs. CG, *p* < .05) t0–t1: depersonalization, no effect t0–t1: personal accomplishment, no effect
Gabbe et al. (2008)	USA, departments of obstetrics and gynecology	Randomized control trial	New chairs (IG;14), (CG;13)	Same as sample	Peer-mentoring-program; individual	Individual	t0: before the intervention; t1: 1 year after the intervention started	Quantitative; Self-rated burnout (Maslach Burnout Inventory, MBI; Maslach and Jackson 1981)	t0–t1: burnout, no effect
Greenberg (2006)	USA, 4 private hospitals	Cohort analytic study; Quasi-experimental manipulation of payment; mixed design with: 2 × 2 (between) X 4 (within)factors, between factors: salary (no change vs. change), intervention (intervention vs. no intervention)	Nursing leaders (IG_total_; 40, IG_underpaid_;19, IG_no payment change;_ 21), (CG; number not mentioned)	Nursing staff members (IG_total_; 241, IG_underpaid_; 105, IG_no payment change_; 136), (CG_total_; 226, CG_underpaid_;96, CG_no payment change_;130)	Group intervention; 2 × 4 h on two consecutive workdays	Organizational justice training (Skarlicki and Latham 2005) with focus on interaction and informational justice	t0: before salary change was announced; t1: after salary change was implemented; t2: 1 week after the intervention; t3: 6 months after the intervention	Quantitative; Self-rated insomnia (adapted version of Jenkins et al. 1996, 1988)	t2: insomnia ↓in IG_underpaid_ (IG_underpaid_ vs. CG_underpaid_) t3: insomnia ↓in IG_underpaid_ (IG_underpaid_ vs. CG_underpaid_)
Haraway and Haraway (2005)	USA, 1 health care organization	Cohort study	Leaders from different professions (IG; 22)	Same as sample	Group intervention; 6 h divided on 2 days 1 week apart	1st day: reasons for conflicts, danger of conflicts, different reactions to conflicts, conflict management, transfer into daily routine 2nd day: reviewing the practical phase, managing difficult people (Bissell 1993), communication skills	t0: before the intervention; t1: 1 month after the intervention	Mixed methods; Self-rated occupational stress (Revised Occupational Stress Inventory OSI-R, three scales with 16 subscales; Osipow 1998) Qualitative analyses to initial situation, expectations for and evaluation of the intervention	t0–t1: subscale role overload ↓ ( *p* < .05) t0–t1: subscale interpersonal strain ↓ ( *p* < .05) t0–t1: subscale role boundary ↓( *p* < .05) t0–t1: subscale psychological strain ↓ ( *p* < .05)
Luk (2018)	Hong Kong, 1 acute general hospital	Cohort study	Senior nursing managers (IG; 42)	Same as sample	Group intervention; 5 × 1.5 h seminar, 5 × 1.5 h small group sharing, 6 h retreat	personal and professional enhancement as a leader through program focusing on servant leadership style	t0: before the intervention t1: after the intervention	Mixed methods; General measure of servant leadership (Ehrhart 2004) Workplace Well-being Questionnaire (WWQ Hyett and Parker 2015,) Content-analysis of self-reflective essays	t0–t1: overall servant leadership ↑ t0–t1: subscales servant leadership: Empowering staff members ↑ Behaving ethically ↑ Having conceptual skills ↑ Creating values for those outside of organization ↑ (all *p* < .05) t0–t1: overall workplace well-being ↑ t0–t1: work well-being subscales: Work satisfaction ↑ Organizational respect for the employee ↑ Employer care ↑ (all *p* < .05)
Stansfeld et al. (2015)	UK, 4 mental health services (NHS Mental Health Trust)	Controlled clinical trial	Leaders from different professions (IG; 49), (CG; 11)	Staff members (IG; 341), (CG; 83)	Mainly online-based intervention; weekly or two weekly over 3 months	e-learning health-promoting program, topics: stress management understanding the link between mental and physical health, leaders’ legal duty of care supporting staff members in problem-solving, find individual staff-centered solutions (Anderson Peak Performance package)	t0: before the intervention; t1: 3 months after the intervention	Mixed methods; Self-rated well-being (Warwick Edinburgh Mental Well-being Scale, WEMWBS Tennant et al. 2007) Sickness absence (with reporting system of NHS mental Health Trust and local Social Service) Self-reported sickness Self-rated psychological distress (General Health Questionnaire, GHQ12 Goldberg and Williams 1988) Interviews with key informants, participating leaders and staff members	t0–t1: no significant effects on mental health
Zimber et al. (2001)	Germany, 11 retirement homes	Controlled clinical trial	Nursing leaders (IG; 24), (CG; 18)	Nursing staff members (IG; 32), (CG; 38)	Group intervention; 12 × 1.5 h weekly	Leaders and staff members: coping with ‘difficult’ residents, professional self-image, coping with stress and personal problems Only leaders: leadership skills and communication with staff members	t0: before the intervention; t1: after the intervention; t2: 3–4 months after the intervention (IG)	Quantitative, Self-rated psychological distress (General Health Questionnaire, GHQ Goldberg and Hillier 1979) Self-rated work atmosphere (Kempe and Closs 1985) Self-rated competence and controlling conviction (Fragebogen zu Kompetenz und Kontrollüberzeugung, FKK Krampen 1991) Self-rated workload in hospitals (Tätigkeits- und Analyseverfahren, TAA-KH Büssing and Glaser 1999) Self-rated professional competence (Zimber and Teufel 1999)	Results for leaders and staff members IG vs. CG: t0–t1: no significant trend differences in mental health outcomes, IG perceived better work atmosphere in relation to residents ↑(*p* = .01) t0–t2: changes in personal competences could predict a significant amount of variance (32%) of working strain and psychological impairment (32%) in regression analysis

*IG* intervention group, *CG* control group

Overall, none of the included studies revealed any adverse effects of a leadership intervention on leaders’ and/or staff members’ mental health. Three studies reported a decrease of negative mental health outcomes (Eastburg et al. 1994; Greenberg 2006; Haraway and Haraway 2005), whereas Luk (2018) showed an increase of well-being in the workplace. In two studies, outcome assessors perceived no significant change in any indicator of mental health (Gabbe et al. 2008; Stansfeld et al. 2015). Zimber et al. (2001) reported a change in a cross-sectional regression analysis but failed to show a trend difference in mental health outcomes.

## Discussion

To our knowledge, this is the first systematic review to evaluate leadership interventions designed to improve the mental health of leaders and/or their staff members in the health care sector. With regard to the research question, this systematic review has three key findings.

First, the seven included studies showed mixed evidence for leadership interventions on mental health (of leaders and/or staff). None of the eligible studies showed an adverse effect on mental health, two studies showed no effect (Gabbe et al. 2008; Stansfeld et al. 2015), one study could not identify a trend difference but found an association between the personal competence and work strain/psychological impairment via regression analysis (Zimber et al. 2001), and the data of four studies suggested a significant positive trend for leaders’ (Haraway and Haraway 2005; Luk 2018) or staff members’ mental health (Eastburg et al. 1994; Greenberg 2006) initiated by a leadership intervention. Second, seven studies could fulfill the search criteria with noticeable diverse research of moderate-to-low quality. Third, no study took place in an ambulatory care setting.

The statistically significant results can be interpreted as clinically relevant, because they all target important interpersonal dimensions for a good relationship between leaders and their staff members in the health care sector which is finally an important factor for a successful patient care (e.g., Boamah et al. 2018), whereas a standardized effect size was only reported by Greenberg (2006) who states a high effect size of the organizational justice intervention.

The diversity of eligible studies was also visible in study sample, intervention type, dose, content, and measurement type. The largest portion (four studies) investigated nursing employees, which is comparable to other research (e.g., Vance and Larson 2002). Leadership interventions differed as well in their direction of action. Three of the studies targeted leaders’ individual mental health as a preventive behavior intervention, whereas two-thirds aimed to improve staff members’ mental health and thus tried to foster mental health through an organizational prevention.

The studies also showed a broad spectrum of intervention types (from basic communication skills to specific models of psychological strain at the workplace), duration and content aspects. The same applies for the measurement instruments, which recorded the full range of mental health (positive as well as negative) from the subjective symptom (insomnia) over subjectively perceived psychological variables (e.g., emotional exhaustion) to objective variables (e.g., sickness absence).

Although studies were diverse, we found some overlapping aspects in effective leadership interventions. Most interventions included educational parts, reflective parts and practical phases where leader could implement their new knowledge in their day-to-day work. Three of four effective interventions used a group setting with the idea of collegial intervision. Contently some effective interventions comprised the communicative handling of difficult situations with staff members (e.g., conflicts or injustice). Following these aspects, an improvement on a behavioral and organizational level could be achieved.

Based on the limitations of these seven studies, we recommend future studies to improve their study design using randomized controlled trials, controlling for confounders by at least conducting studies over more than one setting (including ambulatory care), using a blinding mechanism to reduce socially desirable response patterns of participants and their staff members, employing longer follow-up periods and extend their study population to increase the power of studies (for an overview, see Skivington et al. 2018).

To examine the effect of different study formats (e.g., individual-based interventions vs. group interventions), intervention contents or dose, a comparison of different intervention arms and control groups such as in psychotherapy research (e.g., Zipfel et al. 2014) could be one way to focus on the effectiveness of leadership interventions. Using these study designs could reveal possibly more evidence-based causal relationships between leadership behavior and the mental health of leaders and their staff members. Consequently, we encourage researchers and stakeholders in the health care sector to investigate existing and new implemented leadership interventions in a controlled design to apply more evidence-based health preventive leadership interventions as these interventions seem to have a promising effect on mental health.

Studies that attempted to improve supportive leadership behavior, even though not focusing specifically on mental health in the health care sector, can support this development. Saravo et al. (2017) investigated an intervention designed to improve transformational and transactional leadership behavior in resident physicians. Compared to the control group, external and self-assessment both showed a significant improvement of supportive leadership skills in the intervention group with a large effect size. Awad et al. (2004) also implemented a leadership program for residents, which improved communication skills in the pre–post comparison. Although the improvement in leadership behavior can be seen as one step, future research must go further and acquire staff members’ and leaders’ mental well-being and mental health to clarify the causal association of leadership behavior and staff members’ mental health longitudinally with subjective outcomes (e.g., questionnaires) and objective outcomes (e.g., sickness absence).

Research in other sectors has taken these attempts one step further. Milligan-Saville et al. (2017) conducted a leadership intervention on mental health knowledge and communication for firefighters in a randomized controlled trial. In the 6-month follow-up period, the work-related sickness absence of the staff members in the intervention group decreased significantly (Milligan-Saville et al. 2017). Although the role of firefighters as first aiders can be seen as parallel to ambulance services, the working context of the health care sector is much broader, and thus results can be a hint but are not generally transferable without caution.

Besides these exemplary studies, a review on leadership intervention promoting mental health without any sector specification could identify five studies that targeted staff members’ mental health directly (Tsutsumi 2011). Tsutsumi (2011) summarized that leadership interventions had a positive effect on staff members’ mental health at least in a 1-year intervention period, whereas long-term effects were not investigated by the reviewed studies. Compared to our systematical review, Tsutsumi (2011) only included studies with a focus on staff members’ mental health and did not include leaders’ mental health, limited the search period to 9 years (2000–2009), and did not follow the PRISMA statement (Liberati et al. 2009).

Moreover, a recent review (Kuehnl et al. 2019) on the association of human resource management training in general and staff members’ mental health only comprises 25 studies with a rather low quality of study design. As a result, the authors suggest a rather low impact of leadership interventions on staff members’ mental health. This can be seen as discrepant to our systematic review, but parallel to our estimate, the authors emphasized the need for well-designed further studies (Kuehnl et al. 2019).

These two reviews show that research and study design of mental health preventive leadership interventions need to improve not only in the health care sector but also independent of the specific working context. Consequently, occupational health research on leaders needs to professionalize and catch up with other branches of research (e.g., psychotherapy research).

To get the results of this systematic review in line with the current occupational prevention research in the health care sector, it is important to analyse other existing organizational and behavior preventive interventions for maintaining/fostering mental health. Although we only identified a small number of scientifically pre–post-evaluated leadership interventions targeting mental health, there are other organizational preventive and behavior preventive approaches, which aim to improve mental health in the health care sector workforce.

Ruotsalainen et al. (2015) investigated in their meta-analysis controlled trials on work-related stress prevention in the health care sector and analysed their evidence along the categories organizational and behavioral-level interventions. The only examined organizational interventions that revealed an effect on employees’ stress in their review were changes in working schedules, which had a low evidence level. Regarding relaxation interventions or cognitive behavioral therapy, these behavior-based interventions led to a decrease of stress in comparison to no intervention. However, these results were classified as low-quality evidence as well (Ruotsalainen et al. 2015).

Leadership interventions have the advantage of providing the opportunity to combine organizational and behavioral preventive contents in one training format. Accordingly, they have the potential to be effective in both preventive ways (behavioral and organizational) at the same time and are consequently an opportunity to foster and maintain employees’ mental health in the health care sector. Yet, essential prerequisites for effective organizational prevention through leadership intervention are an unconditional support e.g., of the hospital management and favorable general conditions with regard to the financing of health care institutions. Leadership interventions can be seen as one puzzle piece of mental health prevention, but staff shortage and financial pressure in the health care sector need to be addressed on a political level.

### Limitations

Although we conducted our review according to the standards of the PRISMA statement (Liberati et al. 2009), we are aware of limitations of this review. Because of terms like ‘communication’ or ‘interaction’, our search strategy remained broad and thus agreement among the screeners was in parts unsatisfactory. Furthermore, only articles in German and English language were included. We also decided to choose a restrictive definition of the outcome criteria, mental health, following the WHO (World Health Organization 2001) instead of a broader definition that included job satisfaction as a predictor of positive mental health (Gregersen et al. 2016). In this way, we kept our PICOS criteria clearly structured but were also aware of the potential loss of leadership interventions with other possible stress-preventive contents. We also decided to include only studies with a pre–post-design. This explains the huge reduction from search hits (11,221) to included studies (7). We accepted this reduction, as we were interested in the change potential of leadership interventions and are aware of the neglecting of cross-sectional studies.

## Conclusions

So far, there exist a small number of scientifically evaluated leadership interventions aiming to foster mental health in the health care sector. When summarizing the evidence basis of these studies, interventions that address leadership seem to be the most promising strategies to address mental health in health care employees. Especially interventions with reflective and interactive parts in group setting at several seminar days seem to be effective. However, leadership interventions for maintaining or fostering mental health can be seen as under-examined, so leadership research with regard to mental health from a behavioral prevention and with a (structural) organizational perspective should be extended with high-quality study designs. This is the basis for meta-analytical approaches to review the effect of leadership interventions aiming to maintain or foster mental health. From a practical point of view, mental health-oriented leadership approaches with a focus on relational competence have the potential to combine organizational and behavioral strategies for the promotion of mental health and should be structurally integrated into the regular education of health care workers (e.g., physicians and nurses). There is a great need for health care leaders who are sensitized for behavioral and organizational approaches to the urgent issue of mental health prevention in hospitals as well in ambulatory care. Especially under the aspect of modern technology and artificial intelligence relational and communicative competences are needed to foster the mental health of employees. However, despite their importance, leadership interventions are no substitute for political action against staff shortages and better general conditions in the health care system.
